# AIEC infection triggers modification of gut microbiota composition in genetically predisposed mice, contributing to intestinal inflammation

**DOI:** 10.1038/s41598-018-30055-y

**Published:** 2018-08-17

**Authors:** Alexis Bretin, Cécily Lucas, Anaïs Larabi, Guillaume Dalmasso, Elisabeth Billard, Nicolas Barnich, Richard Bonnet, Hang Thi Thu Nguyen

**Affiliations:** 1 M2iSH (Microbes, intestine, inflammation and Susceptibility of the Host), UMR 1071 Inserm, Université Clermont Auvergne, INRA USC 2018, Clermont-Ferrand, 63001 France; 2 Centre Hospitalier Universitaire (CHU), Clermont-Ferrand, 63001 France

## Abstract

A high prevalence of adherent-invasive *E*. *coli* (AIEC) in the intestinal mucosa of Crohn’s disease patients has been shown. AIEC colonize the intestine and induce inflammation in genetically predisposed mouse models including CEABAC10 transgenic (Tg) mice expressing human CEACAM6-receptor for AIEC and *eif2ak4*^−/−^ mice exhibiting autophagy defect in response to AIEC infection. Here, we aimed at investigating whether gut microbiota modification contributes to AIEC-induced intestinal inflammation in these mouse models. For this, *eif2ak4*^+/+^ and *eif2ak4*^−/−^ mice or CEABAC10 Tg mice invalidated for *Eif2ak4* gene (Tg/*eif2ak4*^−/−^) or not (Tg/*eif2ak4*^+/+^) were infected with the AIEC reference strain LF82 or the non-pathogenic *E*. *coli* K12 MG1655 strain. In all mouse groups, LF82 colonized the gut better and longer than MG1655. No difference in fecal microbiota composition was observed in *eif2ak4*^+/+^ and *eif2ak4*^−/−^ mice before infection and at day 1 and 4 post-infection. LF82-infected *eif2ak4*^−/−^ mice exhibited altered fecal microbiota composition at day 14 and 21 post-infection and increased fecal lipocalin-2 level at day 21 post-infection compared to other groups, indicating that intestinal inflammation developed after microbiota modification. Similar results were obtained for LF82-infected Tg/*eif2ak4*^−/−^ mice. These results suggest that in genetically predisposed hosts, AIEC colonization might induce chronic intestinal inflammation by altering the gut microbiota composition.

## Introduction

The human gastrointestinal tract is inhabited by 10^13^–10^14^ microorganisms, which together make up the intestinal microbiota^1^. The intestinal microbiota is in a symbiotic relationship with its  host and is involved in host nutrition metabolism, maintenance of structural integrity of the gut mucosal barrier, immunomodulation, and protection against pathogens^2^. Thus, an alteration in the composition of the intestinal microbiota, or dysbiosis, is associated with an increased risk of developing diseases such as diabetes, obesity, or chronic diseases of the intestinal tract including ulcerative colitis, Crohn’s disease (CD), celiac disease and irritable bowel syndrome^3^.

CD is a multifactorial disease, of which the etiology involves environmental, genetic, immunological and infectious factors^4^. Intestinal dysbiosis has been observed in CD patients, with most studies have shown reduced diversity of the gut microbiota, a decrease in *Firmicutes* and an increase in *Proteobacteria* and *Bacteroidetes*^5–11^. In addition, evidence has shown an increase in intestinal mucosa-associated *Escherichia coli* in CD patients relative to control subjects, in particular adherent-invasive *E*. *coli* (AIEC) strains with specific pathogenic characteristics^12–20^. The prevalence of the  AIEC strains is increased in ileal lesions of CD patients (36–52%) compared to control subjects (6–17%)^14,16,17,21^. The characterization of the AIEC strains has shown that they are able to adhere to and invade intestinal epithelial cells^22^, survive and multiply in macrophages^23^, and to induce a strong proinflammatory response^23^. In genetically susceptible CEABAC10 transgenic (Tg) mice expressing the human CEACAM6, which is abnormally expressed in ileal epithelial cells of CD patients^24^, AIEC colonize the gut and induce intestinal inflammation^25^.

The multiplication of AIEC inside host cells has been shown to be controlled by autophagy, a homeostatic process responsible for the elimination of damaged cytoplasmic components or intracellular pathogens  via the lysosomal pathway^26–29^. Our recent study showed that the EIF2AK4-EIF2A-ATF4 signaling pathway is induced in intestinal epithelial cells following infection with AIEC, activating autophagy to control AIEC intracellular replication and to inhibit AIEC-induced inflammation^30^. EIF2AK4 (eukaryotic translation initiation factor 2 alpha kinase 4) belongs to a family of protein kinases that phosphorylate the alpha subunit of EIF2 (eukaryotic translation initiation factor 2) in response to various stress stimuli. Phosphorylation of EIF2A in response to EIF2AK4 activation subsequently increases translation of specific mRNAs that have been known to be important for stress remediation such as the activating transcription factor 4 (ATF4)^31^. We showed that the role of the EIF2AK4-EIF2A-ATF4 pathway in autophagy activation in response to AIEC infection is mediated via the ATF4-dependent induced transcription of several autophagy genes^30^. *eif2ak4*^−/−^ mice infected with the AIEC reference strain LF82 exhibit a defect in the induction of autophagy gene transcription and autophagy response in enterocytes, which are accompanied with increased intestinal colonization by LF82 bacteria and aggravated inflammation compared to wild type mice^30^. In addition, studies have begun to show the importance of EIF2AK4 in CD. Indeed, an increase in the expression of EIF2AK4 in patients with CD compared to the controls has been reported^32–34^. Our group also showed activation of the EIF2AK4-EIF2A-ATF4 pathway in ileal biopsies from patients with non-inflamed CD compared to control subjects. However, this was not observed in ileal biopsies from patients with inflamed CD, suggesting that a defect in activation of this pathway could be one of the mechanisms contributing to the active disease^30^.

As gut microbiota has been implicated in the etiopathogenesis of CD, we aimed at investigating whether it is associated with the AIEC-induced intestinal inflammation in genetically predisposed mouse models with *Eif2ak4* gene deficiency.

## Results

### *Eif2ak4* gene deficiency has no impact on the gut microbiota composition of mice

**Figure 1:**
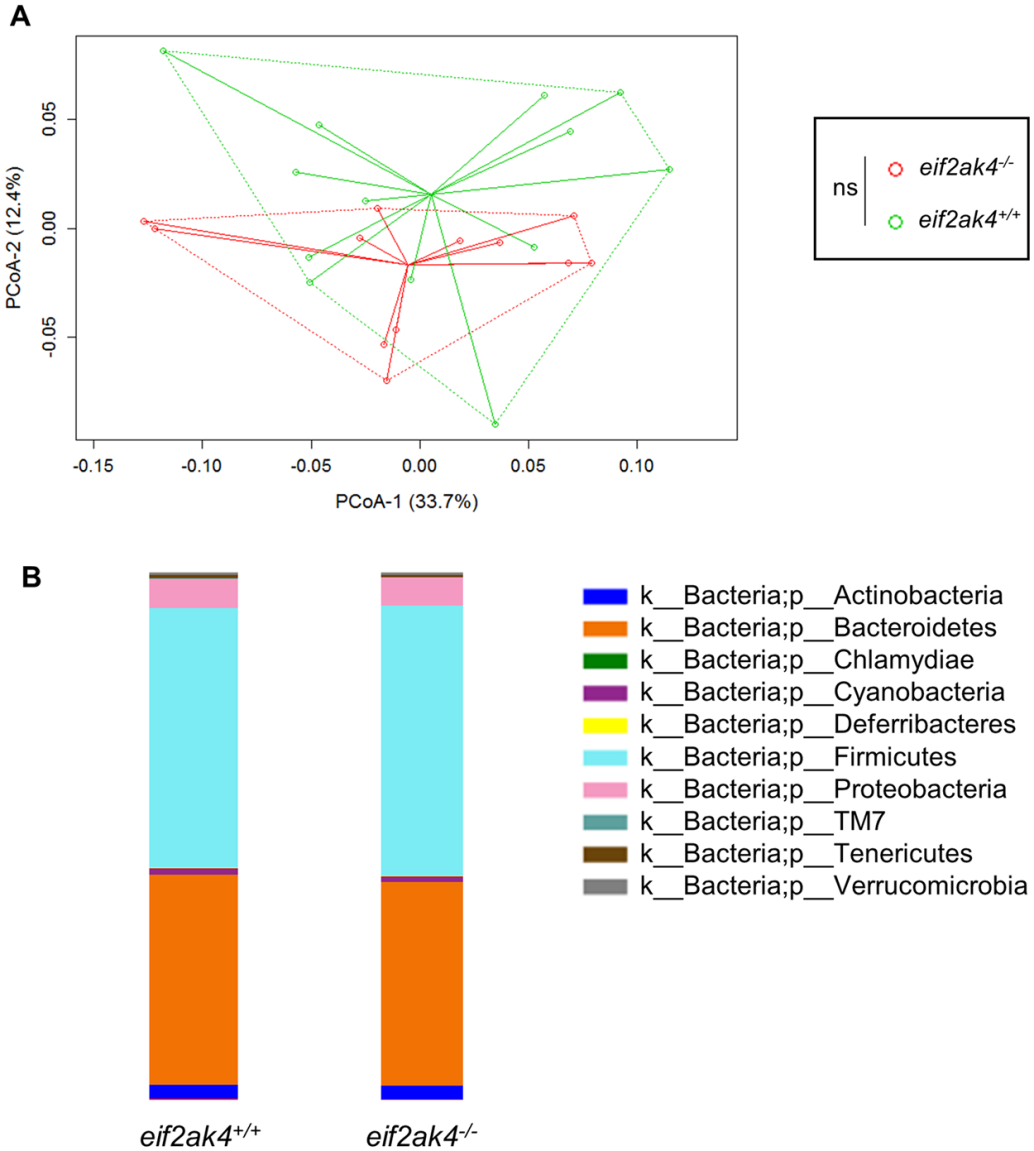
*Eif2ak4* gene deficiency has no impact on the gut microbiota composition of mice. *eif2ak4*^+/+^ (N = 13) and *eif2ak4*^−/−^ (N = 13) mice were housed in specific-pathogen free conditions. Feces were collected from 12-week old mice for the analysis of the bacterial microbiota composition based on Illumina sequencing of the 16S rRNA gene. (**A**) Mouse fecal bacterial communities were clustered using PCoA of the weighted UniFrac distance matrix. PCoA-1 and PCoA-2 were plotted, and the percentage of the variation explained by the plotted principal coordinates was indicated in the Y-X-axis labels. Groups were compared using permanova method. Ns, not significant. (**B**) Relative abundance of phyla in fecal samples from *eif2ak4*^+/+^ and *eif2ak4*^−/−^ mice.

To verify whether *Eif2ak4* gene deficiency could has an impact on the gut microbiota composition, we used wild type (*eif2ak4*^+/+^) and *Eif2ak4*-deficient (*eif2ak4*^−/−^) mice, which were housed in specific pathogen-free conditions. Mice were 12 weeks old as it was previously shown that mice acquire a relatively stable gut microbiota at this age^35^. Fecal samples were collected before any treatment (Day 0) from *eif2ak4*^+/+^ and *eif2ak4*^−/−^ mice, and fecal microbiota analysis was performed by 16S rRNA gene sequencing using Illumina technology. Sequence data were analyzed via the QIIME (quantification insight into microbial ecology) data processing pipeline. Analysis at the genus level by weighted UniFrac and PCoA (principal coordinate analysis) indicated that there was not any pattern of clustering between *eif2ak4*^+/+^ and *eif2ak4*^−/−^ mice (Fig. 1A). Taxonomic analysis showed a high prevalence of bacteria from the *Firmicutes* and *Bacteroidetes* phyla in the gut microbiota of *eif2ak4*^+/+^ and *eif2ak4*^−/−^ mice without any significant difference between these two groups (Fig. 1B). This result showed that the deletion of *Eif2ak4* gene in mice does not induce any change in the composition of the gut microbiota.

### Colonization of *eif2ak4*^−/−^ mice with AIEC results in a significant modification of the gut microbiota composition and triggers intestinal inflammation

In a previous study, we demonstrated that the EIF2AK4/EIF2A/ATF4 pathway is involved in the autophagy-mediated control of AIEC intracellular replication and inflammatory response, and that AIEC infection of *eif2ak4*^−/−^ mice triggers intestinal inflammation^30^. As gut microbiota has been implicated in the etiopathogenesis of CD^4^, we investigated whether it contributes to intestinal inflammation triggered by AIEC infection in *eif2ak4*^−/−^ mice.

In our previous studies^25,30^, the mouse models of AIEC infection involved the treatment of mice with an antibiotic cocktail before AIEC infection to facilitate gut colonization by AIEC. However, as it has been shown that antibiotic use has a huge impact on gut microbiota, we decided to not use any such compound. *eif2ak4*^+/+^ and *eif2ak4*^−/−^ mice were divided into sub groups that were challenged with 10^9^ colony-forming units (CFU) of the AIEC reference strain LF82 or the non-pathogenic *E*. *coli* K12 MG1655 strain or vehicle (PBS) by gavage for 3 days. The mice were then placed in specific pathogen-free housing condition, and the feces were collected at different time points post-infection and used to evaluate inflammation by quantifying lipocalin (lcn)-2 level and to analyze the gut microbiota composition by sequencing the 16S rRNA gene using the Illumina technology.

**Figure 2:**
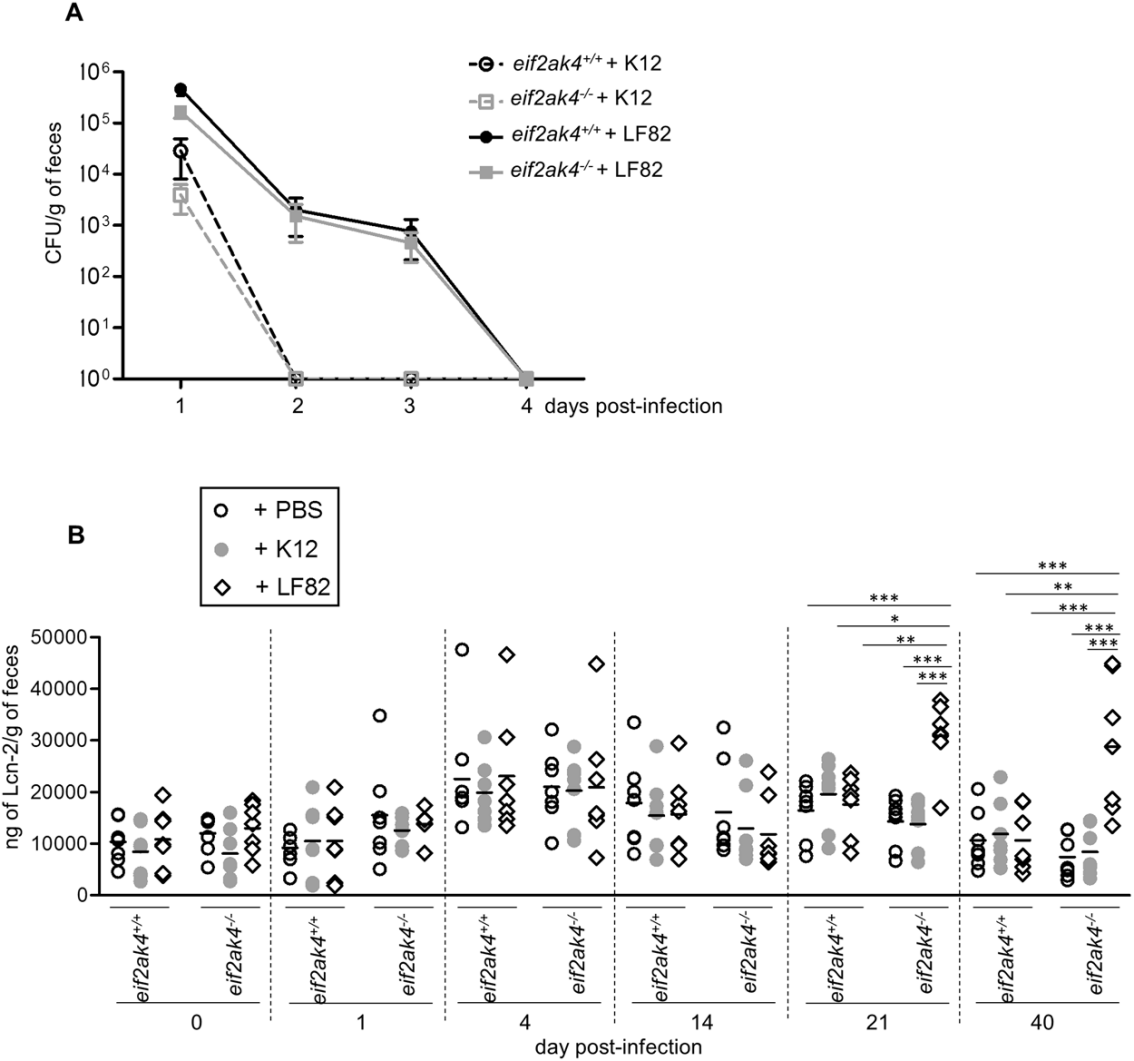
Colonization of *eif2ak4*^−/−^ mice with AIEC triggers chronic gut inflammation. *eif2ak4*^+/+^ and *eif2ak4*^−/−^ mice were challenged by oral gavage for 3 days (once per day) with PBS (N = 6 mice per group) or with 10^9^ CFU of the AIEC LF82 strain (N = 7 mice per group) or the non-pathogenic *E*. *coli* K12 MG1655 strain (N = 6 mice per group). (**A**) The numbers of AIEC LF82 and *E*. *coli* K12 were determined in the feces collected every day post-infection by platting on LB media containing selective antibiotics. Data are represented as means ± SEM. (**B**) Fecal lcn-2 levels were measured by ELISA. Means were shown as lines. Statistical analysis was performed using one-way ANOVA test followed by Bonferroni’s post-hoc comparisons. *P < 0.05; **P ≤ 0.01; ***P ≤ 0.001.

First, we determined the bacterial CFU in mouse feces, which reflect bacterial persistence in the gut. In both *eif2ak4*^+/+^ and *eif2ak4*^−/−^ mice, the colonization of AIEC LF82 bacteria was higher and lasted longer compared to that of the non-pathogenic K12 MG1655 bacteria as the LF82 strain was detectable up to day 4 post-infection, whereas the K12 MG1655 strain was cleared since day 1 post-infection (Fig. 2A). Furthermore, the increased gut colonization by AIEC in *eif2ak4*^−/−^ mice compared to *eif2ak4*^+/+^ mice as we previously reported using a model of infection following antibiotic treatment^30^ was not observed in the condition of this study (Fig. 2A).

Secondly, we quantified the fecal lcn-2 level, which has been shown as a marker of intestinal inflammation in several mouse models^36^. The results showed no difference in fecal lcn-2 levels in *eif2ak4*^+/+^ mice upon AIEC LF82 or K12 MG1655 infection up to 40 days post-infection (Fig. 2B). However, in *eif2ak4*^−/−^ mice, the fecal lcn-2 level was significantly increased at day 21 and 40 post-LF82 infection, and this was not observed for K12 MG1655 infection (Fig. 2B). Importantly, the increased lcn-2 level in LF82-infected *eif2ak4*^−/−^ mice lasted for at least twenty days, suggesting that a chronic inflammation is set up in *eif2ak4*^−/−^ mice after AIEC infection.

**Figure 3:**
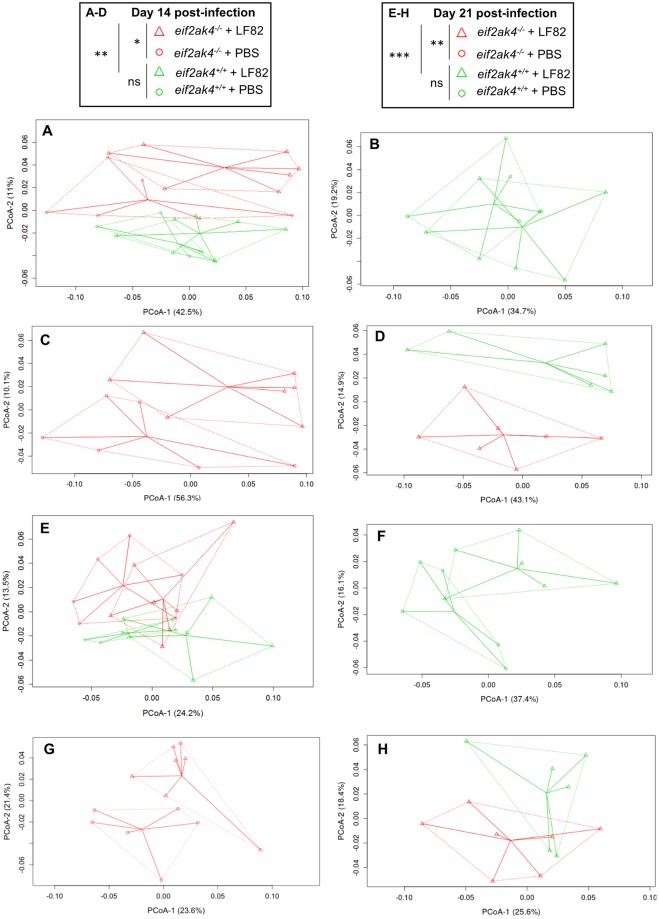
Colonization of *eif2ak4*^−/−^ mice with AIEC results in modification of the gut microbiota composition at day 14 and 21 post-infection. *eif2ak4*^+/+^ and *eif2ak4*^−/−^ mice were challenged by oral gavage for 3 days (once per day) with PBS (N = 6 mice per group) or with 10^9^ CFU of the AIEC LF82 strain (N = 7 mice per group). Feces were collected at day 14 (**A**–**D**) and 21 (**E**–**H**) post-infection for the analysis of the bacterial microbiota composition based on Illumina sequencing of the 16S rRNA gene. Mouse fecal bacterial communities were clustered using PCoA of the weighted UniFrac distance matrix. PCoA-1 and PCoA-2 were plotted, and the percentage of the variation explained by the plotted principal coordinates was indicated in the Y-X-axis labels. (**A**,**E**) Comparison between mouse groups: *eif2ak4*^+/+^ + PBS, *eif2ak4*^+/+^ + LF82, *eif2ak4*^−/−^ + PBS and *eif2ak4*^−/−^ + LF82. (**B**,**F**) Comparison between mouse groups: *eif2ak4*^+/+^ + PBS and *eif2ak4*^+/+^ + LF82. (**C**,**G**) Comparison between mouse groups: *eif2ak4*^−/−^ + PBS and *eif2ak4*^−/−^ + LF82. (**D**,**H**) Comparison between mouse groups: *eif2ak4*^+/+^ + LF82 and *eif2ak4*^−/−^ + LF82. Groups were compared using permanova method. Ns, not significant; *P < 0.05; **P ≤ 0.01; ***P ≤ 0.001.

**Figure 4:**
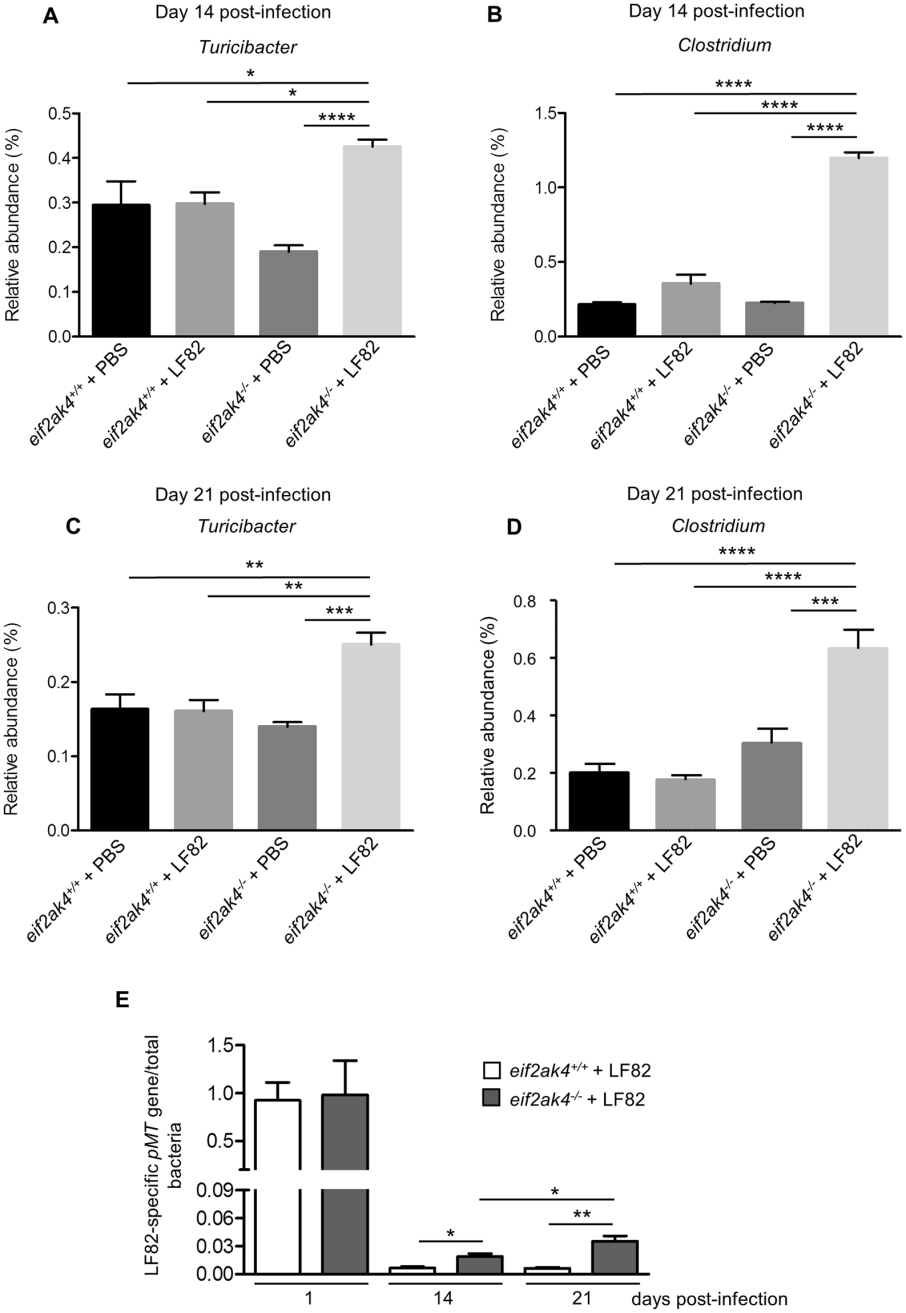
Colonization of *eif2ak4*^−/−^ mice with AIEC results in modification of *Turicibacter* and *Clostridium* abundance at day 14 and 21 post-infection. *eif2ak4*^+/+^ and *eif2ak4*^−/−^ mice were challenged by oral gavage for 3 days (once per day) with PBS (N = 6 mice per group) or with 10^9^ CFU of the AIEC LF82 strain (N = 7 mice per group). Feces were collected at day 14 (**A**,**B**) and 21 (**C**,**D**) post-infection for the analysis of the bacterial microbiota composition based on Illumina sequencing of the 16S rRNA gene. Relative abundance of *Turicibacter* (**A**,**C**) and *Clostridium* (**B**,**D**) between mouse groups: *eif2ak4*^+/+^ + PBS, *eif2ak4*^+/+^ + LF82, *eif2ak4*^−/−^ + PBS and *eif2ak4*^−/−^ + LF82. (**E**) qPCR analysis of the LF82-specific *pMT* gene expression level in the feces at the indicated time. Data were normalized to total bacterial. Fold-induction was calculated using the ΔΔCt method. Values were expressed as means ± SEM. Statistical analysis was performed using one-way ANOVA test followed by Bonferroni’s post-hoc comparisons. Ns, not significant; *P < 0.05; **P ≤ 0.01; ***P ≤ 0.001; ****P ≤ 0.0001.

Next, we analyzed the fecal microbiota composition in these mouse groups at the same time post-infection to investigate whether an altered microbiota composition could occur before or after the increase in lcn-2 level in LF82-infected *eif2ak4*^−/−^ mice. Because colonization with the K12 MG1655 strain did not induce any inflammation in either *eif2ak4*^+/+^ or *eif2ak4*^−/−^ mice, we decided to remove the K12 condition from the microbiota composition analysis. Fecal samples collected over time from *eif2ak4*^+/+^ and *eif2ak4*^−/−^ mice that had been challenged with AIEC LF82 or PBS were subjected to Illumina sequencing of the 16S rRNA genes. Sequence data was analyzed via the QIIME data processing pipeline. Analysis at the genus level by weighted UniFrac and PCoA did not show any pattern of clustering in any of the 4 mouse groups: *eif2ak4*^+/+^ + LF82, *eif2ak4*^−/−^ + LF82, *eif2ak4*^+/+^ + PBS, *eif2ak4*^−/−^ + PBS at day 1 and 4 post-infection (Fig. S1). In contrast, after 14 and 21 days of infection, a clear pattern of clustering in *eif2ak4*^−/−^ + LF82 group compared to the other groups (*eif2ak4*^+/+^ + LF82, *eif2ak4*^+/+^ + PBS and *eif2ak4*^−/−^ + PBS) was observed (Fig. 3). In detail, at day 14 and 21 post-infection, two dimensional (2D) plotting of PCoA-1 *versus* PCoA-2 failed to show a pattern of clustering between *eif2ak4*^+/+^ + PBS and *eif2ak4*^+/+^ + LF82 groups (Fig. 3B,F), indicating that colonization by AIEC may not alter microbiota composition in wild type host. In contrast, AIEC LF82 colonization resulted in altered microbiota composition in *eif2ak4*^−/−^ mice at days 14 and 21 post-AIEC administration. 2D plotting of PCoA-1 *versus* PCoA-2 showed a clear difference in the microbiota composition between *eif2ak4*^−/−^ + PBS and *eif2ak4*^−/−^ + LF82 groups at day 14 (Fig. 3C) and 21 (Fig. 3G) post-infection. Furthermore, 2D plotting of PCoA-1 *versus* PCoA-2 also showed a difference in the gut microbiota composition between *eif2ak4*^+/+^ and *eif2ak4*^−/−^ mice after 14 (Fig. 3D**)** or 21 (Fig. 3H**)** days of AIEC administration. Taxonomic analysis at the genus level showed that most of the differences observed in PCoA were explained by an increase in *Turicibacter* and *Clostridium* in *eif2ak4*^−/−^ + LF82 group compared to the other groups at days 14 (Fig. 4A,B) and 21 (Fig. 4C,D) post-infection. These results show that neither *Eif2ak4* gene deficiency nor AIEC colonization alone is sufficient to induce a modification in the gut microbiota composition. However, the combination of the genetic and infectious factors leads to a significant shift in the gut microbiota of the mice. Interestingly, this shift occurred at day 14 post-infection before the appearance of inflammation at day 21 post-infection.

In an effort to explain the change in gut microbiota composition in *eif2ak4*^−/−^ + LF82 group, we performed qPCR to quantify AIEC LF82-specific *pMT* gene expression using specific primers as previously described^37^. Interestingly, qPCR data showed *pMT* gene amplification at day 14 and 21 post-infection in both *eif2ak4*^+/+^ and *eif2ak4*^−/−^ groups, and the *pMT *gene expression level was increased in *eif2ak4*^−/−^ mice compared to *eif2ak4*^+/+^ mice (Fig. 4E). In addition, LF82-specific *pMT* gene expression level in *eif2ak4*^−/−^ mice was increased at day 21 post-infection compared to that at day 14 post-infection (Fig. 4E). However, in *eif2ak4*^+/+^ mice, this level was not increased at day 21 compared to day 14 post-infection (Fig. 4E). This suggests that LF82 bacteria create a colonization niche in *eif2ak4*^−/−^ mice, promoting their expansion at later time.

Together, these data suggest that AIEC colonization in the genetically predisposed host with *Eif2ak4* gene deficiency induces a change in the gut microbiota composition, which is associated with the development of chronic intestinal inflammation.

### AIEC colonization in CEABAC10 transgenic mice deficient in *Eif2ak4* gene induces a modification of the gut microbiota composition and triggers intestinal inflammation

As AIEC colonize the gut and induce intestinal inflammation in CEABAC10 transgenic (Tg) mice expressing human CEACAM6^25^, host epithelial receptor for AIEC adherence^24^, we sought to verify our results in CEABAC10 Tg mice with *Eif2ak4* gene deficiency. For that, we generated Tg/*eif2ak4*^+/+^ and Tg/*eif2ak4*^−/−^ mice and infected them with the AIEC LF82 strain or the K12 MG1655 strain using the same infection protocol as applied for *eif2ak4*^+/+^ and *eif2ak4*^−/−^ mice, in which antibiotics were not used. Feces were collected at different time points post-infection and used to evaluate inflammation by quantifying lcn-2 level and to analyze the gut microbiota composition by Illumina sequencing of the 16S rRNA gene.

**Figure 5:**
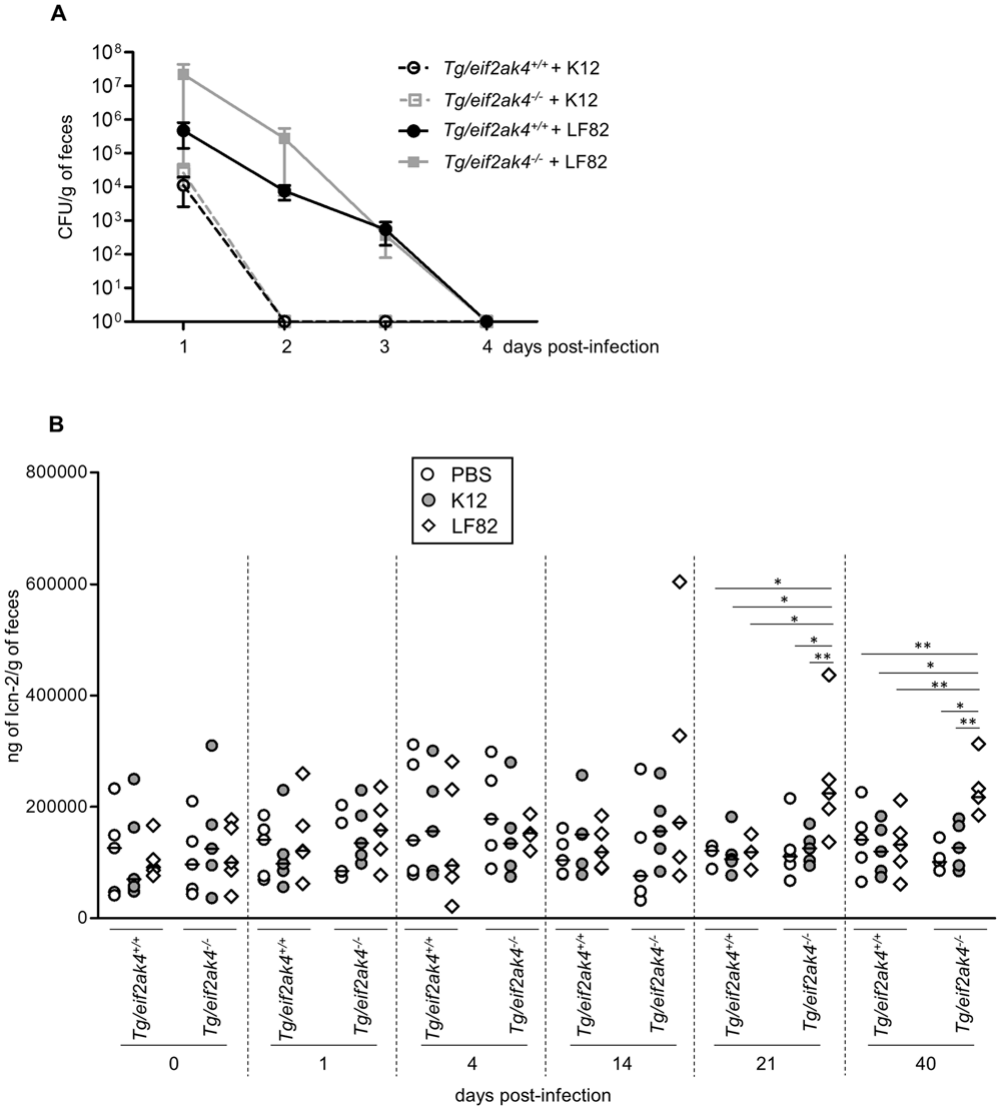
Colonization of CEABAC10 transgenic (Tg) mice deficient in *Eif2ak4* gene with AIEC triggers chronic gut inflammation. Tg/*eif2ak4*^+/+^ and Tg/*eif2ak4*^−/−^ mice were challenged by oral gavage for 3 days (once per day) with PBS or with 10^9^ CFU of the AIEC LF82 strain or the non-pathogenic *E*. *coli* K12 MG1655 strain (N = 5 mice per group). (**A**) The number of AIEC LF82 bacteria was determined in the feces collected every day post-infection by platting on LB media containing selective antibiotics. (**B**) Fecal lcn-2 levels were measured by ELISA. Means were shown as lines. Statistical analysis was performed using one-way ANOVA test followed by Bonferroni’s post-hoc comparisons. *P < 0.05; **P ≤ 0.01.

First, we quantified bacterial CFU in mouse feces, which reflect bacterial persistence in the gut. In both Tg/*eif2ak4*^+/+^ and Tg/*eif2ak4*^−/−^ mice, LF82 bacteria were detectable in the feces up to day 4 post-infection, with no significant difference between the two groups (Fig. 5A). The K12 MG1655 strain was cleared since day 1 post-infection in both Tg/*eif2ak4*^+/+^ and Tg/*eif2ak4*^−/−^ mice (Fig. 5A).

Next, fecal lcn-2 level quantification did not show any difference in Tg/*eif2ak4*^+/+^ mice upon AIEC LF82 infection up to 40 days post-infection (Fig. 5B). However, in Tg/*eif2ak4*^−/−^ mice, a significant increase in fecal lcn-2 level upon AIEC LF82 infection was observed at day 21 post-infection, and this lasted up to day 40 post-infection (Fig. 5B). Although it was performed in two independent experiments, the fecal lcn-2 level of Tg/*eif2ak4*^−/−^ mice (Fig. 5B) appeared to significantly higher than that of *eif2ak4*^−/−^ mice (Fig. 2B), around 10 times. This suggests that Tg mice may favor AIEC-induced inflammation. Infection of both Tg/*eif2ak4*^+/+^ and Tg/*eif2ak4*^−/−^ with the K12 MG1655 strain did not induce any significant change in fecal lcn-2 level up to 40 days post-infection (Fig. 5B). These results were consistent with the results obtained with *eif2ak4*^+/+^ and *eif2ak4*^−/−^ mice, indicating that AIEC colonization triggers a chronic inflammation response in a genetically predisposed host deficient in *Eif2ak4* gene.

**Figure 6:**
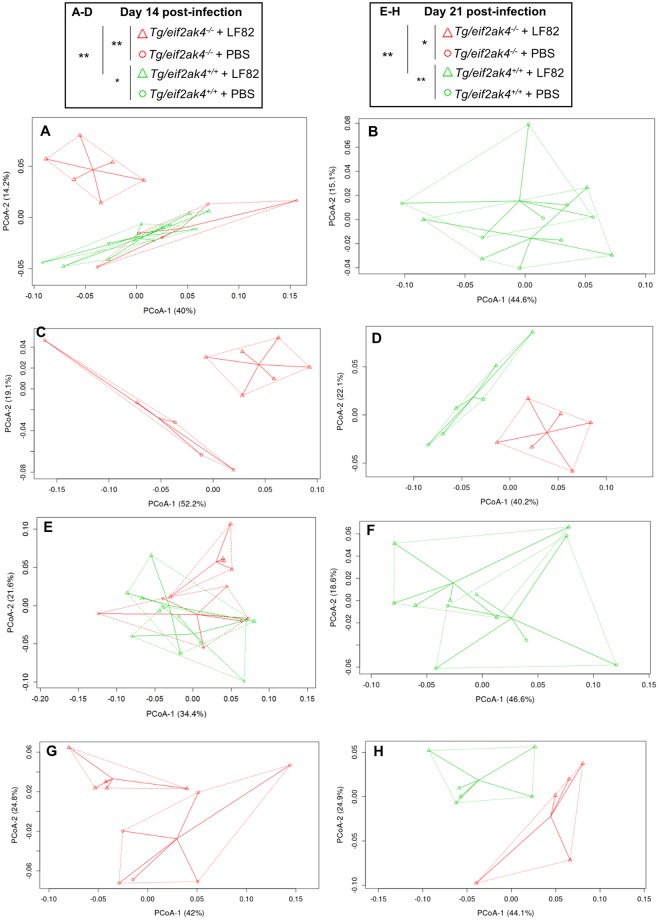
Colonization of CEABC10 transgenic (Tg) mice deficient in *Eif2ak4* gene with AIEC LF82 results in modification of the gut microbiota composition at days 14 and 21 post-infection. Tg/*eif2ak4*^+/+^ and Tg/*eif2ak4*^−/−^ mice were challenged by oral gavage for 3 days (once per day) with PBS (N = 6 mice per group) or with 10^9^ CFU of the AIEC LF82 strain (N = 6 mice per group). Feces were collected at days 14 (**A**–**D**) and 21 (**E**–**H**) post-infection for the analysis of the bacterial microbiota composition based on Illumina sequencing of the 16S rRNA gene. (**A**–**H**) Mouse fecal bacterial communities were clustered using PCoA of the weighted UniFrac distance matrix. PCoA-1 and PCoA-2 were plotted, and the percentage of the variation explained by the plotted principal coordinates was indicated in the Y-X-axis labels. Groups were compared using permanova method. Ns, not significant; *P < 0.05; **P ≤ 0.01. (**A**,**E**) Comparison between mouse groups: *Tg*/*eif2ak4*^+/+^ + PBS, *Tg*/*eif2ak4*^+/+^ + LF82, *Tg*/*eif2ak4*^−/−^ + PBS and *Tg*/*eif2ak4*^−/−^ + LF82. (**B**,**F**) Comparison between mouse groups: *Tg*/*eif2ak4*^+/+^ + PBS and *Tg*/*eif2ak4*^+/+^ + LF82. (**C**,**G**) Comparison between mouse groups: *Tg*/*eif2ak4*^−/−^ + PBS and *Tg*/*eif2ak4*^−/−^ + LF82. (**D**,**H**) Comparison between mouse groups: *Tg*/*eif2ak4*^+/+^ + LF82 and *Tg*/*eif2ak4*^−/−^ + LF82.

**Figure 7:**
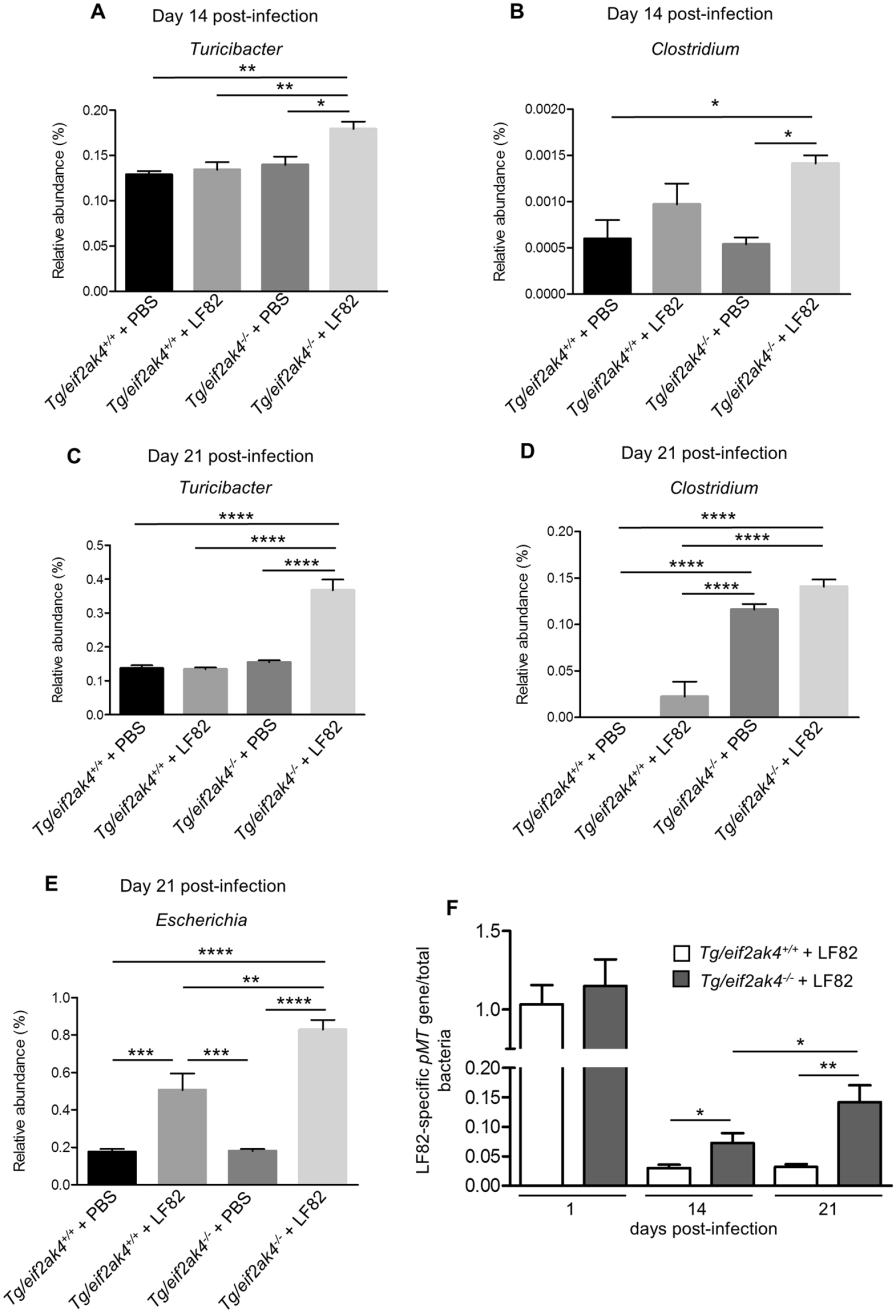
Colonization of Tg/*eif2ak4*^−/−^ mice with AIEC results in modification of *Turicibacter*, *Clostridium* and *Escherichia* abundance. Tg/*eif2ak4*^+/+^ and Tg/*eif2ak4*^−/−^ mice were challenged by oral gavage for 3 days (once per day) with PBS (N = 6 mice per group) or with 10^9^ CFU of the AIEC LF82 strain (N = 6 mice per group). Feces were collected at day 14 (**A**,**B**) and 21 (**C**–**E**) post-infection for the analysis of the bacterial microbiota composition based on Illumina sequencing of the 16S rRNA gene. Relative abundance of *Turicibacter* (**A**,**C**), *Clostridium* (**B**,**D**) and *Escherichia* **(E)** between mouse groups: Tg/*eif2ak4*^+/+^ + PBS, Tg/*eif2ak4*^+/+^ + LF82, Tg/*eif2ak4*^−/−^ + PBS and Tg/*eif2ak4*^−/−^ + LF82. (**F**) qPCR analysis of the LF82-specific *pMT* gene expression level in the feces at the indicated time. Data were normalized to total bacterial. Fold-induction was calculated using the ΔΔCt method. Values were expressed as means ± SEM. Statistical analysis was performed using one-way ANOVA test followed by Bonferroni’s post-hoc comparisons. *P < 0.05; **P ≤ 0.01; ***P ≤ 0.001; ****P ≤ 0.0001.

Finally, microbiota composition analysis using fecal samples from Tg/*eif2ak4*^+/+^ and Tg/*eif2ak4*^−/−^ mice before any treatment (day 0) by weighted UniFrac and PCoA did not show any pattern of clustering between the two genotypes (Fig. S2). At day 1 and 4 post-infection, we did not observe any pattern of clustering between uninfected (+PBS) or LF82-infected Tg/*eif2ak4*^+/+^ and Tg/*eif2ak4*^−/−^ groups (Fig. S3). In contrast, at day 14 and 21 post-infection, patterns of clustering between *eif2ak4*^−/−^  + LF82, *eif2ak4*^+/+^  + LF82, *eif2ak4*^+/+^  + PBS and *eif2ak4*^−/−^  + PBS groups were observed (Fig. 6A,E). Interestingly, in Tg/*eif2ak4*^+/+^ mice, in contrast to *eif2ak4*^+/+^ mice, the microbiota of uninfected and LF82-infected groups diverged at day 14 and 21 post-infection (Fig. 6B,F). As it was shown for *eif2ak4*^−/−^ mice, AIEC colonization also induced a change in the microbiota composition in Tg/*eif2ak4*^−/−^ mice. These changes were readily apparent by 14 and 21 days post-infection, as 2D plotting of PCoA-1 *versus* PCoA-2 showed a shift in the gut microbiota composition of Tg/*eif2ak4*^−/−^ mice compared to the other groups (Fig. 6C,G). Furthermore, 2D plotting of PCoA-1 *versus* PCoA-2 of Tg/*eif2ak4*^+/+^ + LF82 and Tg/*eif2ak4*^−/−^  + LF82 groups also showed a significant difference in the gut microbiota between these two groups (Fig. 6D,H). Taxonomic analyses at the genus level were consistent with those performed with *eif2ak4*^+/+^ and *eif2ak4*^−/−^ mice as most of the differences observed in PCoA were explained by an increase in *Turicibacter* and *Clostridium* in Tg/*eif2ak4*^−/−^+ LF82 group compared to the other groups (Fig. 7A–D). Nevertheless, we observed an increase of *Escherichia* in Tg/*eif2ak4*^−/−^  + LF82 group compared to the other groups at day 21 post-infection (Fig. 7E), and this was not observed for *eif2ak4*^−/−^ + LF82 mice).

In addition, we determined AIEC LF82 colonization by quantifying *pMT* gene expression by qPCR at day 14 and 21 post-infection in both Tg/*eif2ak4*^−/−^ and Tg/*eif2ak4*^+/+^ groups. LF82-specific *pMT* gene expression level was increased in Tg/*eif2ak4*^−/−^ compared to Tg/*eif2ak4*^+/+^ mice at day 14 and 21 post-infection, although no significant difference was detected at day 1 post-infection (Fig. 7F). In addition, in Tg/*eif2ak4*^−/−^ + LF82 mice, pMT gene expression level was increased at day 21 compared to day 14 post-infection, and this was not observed for Tg/*eif2ak4*^+/+^ mice (Fig. 7F). This suggests that LF82 bacteria can persist in the gastrointestinal tract of Tg/*eif2ak4*^−/−^ mice and expanse their colonization at later time.

These results, which were consistent with those obtained with non-transgenic mice, confirm that AIEC colonization in a genetically predisposed host with *Eif2ak4* gene deficiency induces a shift in the gut microbiota composition, which may select a pro-inflammatory microbiota, and this is accompanied with chronic gut inflammation.

## Discussion

Extensive studies have suggested that the etiology of CD involves environmental and genetic factors that lead to dysfunction of the intestinal epithelial barrier with consequent deregulation of the mucosal immune system and responses to gut microbiota. The AIEC strains, which abnormally colonize the ileal mucosa of CD patients, have emerged as “pathobionts” implied in the pathogenesis of CD^4^.

Our recent study showed that the EIF2AK4-EIF2A-ATF4 signaling pathway is induced in intestinal epithelial cells following infection with AIEC, leading to autophagy activation to control AIEC intracellular replication and to inhibit AIEC-induced inflammation^30^. *eif2ak4*^−/−^ mice exhibit a defect in autophagy response to AIEC infection, accompanied with increased intestinal colonization by AIEC and aggravated inflammation compared to wild type mice. In the current study, we aimed at investigating whether a modification in the gut microbiota composition could contribute to the AIEC-induced intestinal inflammation in genetically predisposed mice with *Eif2ak4* gene deficiency.

In the past decades, numerous studies have tried to show a role for the gut microbiota in the development of CD. Indeed, it has been shown that a treatment with antibiotics such as ciprofloxacin or metronidazole is efficient to treat CD patients^38^. It has also been shown that antibiotic use in early childhood is a risk factor for inflammatory bowel disease development in adults^39^. It has become evident that CD patients exhibit intestinal dysbiosis with a decrease in the number of potentially beneficial bacteria such as *Bifidobacteria*, *Lactobacilli* and *Firmicutes* and an increase in that of putative pathogenic bacteria such as *Bacteroides* and *Escherichia coli*^4^. The key role of the gut microbiota trigger in CD development has also been shown in mouse models. For example, the TNF^deltaARE^ mice carrying a deletion in the TNF AU (adenosin-uracil)-rich elements (ARE) develop transmural inflammation in the distal ileum that shares some clinical features with human CD only after colonization with specific-pathogen free microbiota, and germ-free TNF^deltaARE^ mice were protected from intestinal inflammation^40^. A modification in the gut microbiota composition, with an increase in *Bacteroidetes* and *E*. *coli*, triggered by a high fat/high sugar diet, has been shown to induce enhanced susceptibility to AIEC infection and intestinal inflammation in CEABAC10 transgenic mice^41,42^. It was also shown that, in mice lacking the bacterial flagellin receptor TLR5 (*tlr5*^−/−^ mice), AIEC colonization induces a chronic inflammation and a gut microbiota modification toward a microbiota that expresses increased lipopolysaccharide and flagellin levels, and these effects lasted even many days after the AIEC clearance^43^.

In the present study, we reported that AIEC colonization triggers a gut microbiota modification in *eif2ak4*^−/−^ mice. This modification comes along with increased abundance of *Clostridium* and *Turicibacter*, which have been shown to be increased under inflammatory condition^44^. In addition, analysis of fecal microbiota composition from 235 patients with inflammatory bowel disease (149 with Crohn’s disease and 86 with ulcerative colitis) and 38 healthy subjects (HS) using 16S rRNA gene sequencing showed an increase in *Turicibacter* in ileum CD group compared to HS group (Kruskal-Wallis test with Dunn’s multiple comparison test, *P < 0.05; data not shown from the study by Sokol *et al*.published in Gut in 2017)^45^. No difference in *Turicibacter* between HS group and colon CD group or ulcerative colitis group was observed^45^. Further investigations are necessary to show whether the elevated *Turicibacter* and *Clostridium* abundance is sufficient to cause intestinal inflammation or contribute to the development of intestinal inflammation in genetically modified mouse models.

In *eif2ak4*^−/−^ mice expressing human CEACAM6 (Tg/*eif2ak4*^−/−^ mice), which mimic abnormal expression of CEACAM6 in ileal epithelial cells in CD^24^, we also observed a modification of gut microbiota composition upon AIEC colonization. This was accompanied with increased abundance of *Escherichia* in Tg/*eif2ak4*^−/−^ + LF82 mice, which was not observed in *eif2ak4*^−/−^ mice colonized with AIEC LF82. It is worthy to note that the *Escherichia* data resulted from Illumina 16S rRNA gene sequencing cannot distinguish between autochthonous *E*. *coli* and AIEC LF82 abundance. This result could be explained by the fact that Tg/*eif2ak4*^−/−^ mice express human CEACAM6, which is the receptor for AIEC to colonize the ileal epithelium^24^, and this receptor could promote *Escherichia* colonization in these mice.

Importantly, AIEC colonization triggers a chronic inflammation in both *eif2ak4*^−/−^ and Tg/*eif2ak4*^−/−^ mice, which lasted for days. Furthermore, inflammation occurred in *eif2ak4*^−/−^ and Tg/*eif2ak4*^−/−^ mice colonized with AIEC after the appearance of gut microbiota modification. These results suggest that the altered microbiota composition in the genetically predisposed hosts colonized with AIEC might contribute to the development of chronic intestinal inflammation. It was previously shown that AIEC colonization of germ-free *tlr5*^−/−^ mice drives chronic inflammation which correlates with microbiota components^43^. This study was performed in germ-free animals, which have been largely used to characterize the specific effects of known strains of bacteria or of the gut microbiota as a whole upon colonization^46^. However, they exhibit defects in the immune system^47^, which is not always the case in genetically predisposed hosts. Herein, we showed the contribution of gut dysbiosis triggered by AIEC colonization in the development of chronic inflammation in genetically predisposed hosts carrying a microbiota, which was not previously shown. Our study also showed that neither the genetic factors (deficiency in *Eif2ak4* gene with or without abnormal human CEACAM6 expression) nor the microbial factor (AIEC colonization) alone is sufficient to induce a modification in the gut microbiota composition, reinforcing the notion of the multifactorial and complex etiology of CD.

The underlying mechanism by which AIEC colonization modifies the gut microbiota composition in *eif2ak4*^−/−^ and Tg/*eif2ak4*^−/−^ mice remains uninvestigated. We hypothesize that a defect in autophagy response in mice with *Eif2ak4* deficiency upon AIEC infection, which was previously shown^30^, may affect immune response to gut microbiota and thereby contribute to gut dysbiosis. In this regard, it has been reported that a defect in autophagy genes, such as *Nod2* or *Atg16l1*, induced a shift in gut microbiota^35,48,49^. Indeed, it has been shown that the bacterial microbiota composition was altered in *Nod2* knockout mice compared to wild type mice^35,48^. This change was present in adult mice but also in weaning mice, indicating a crucial role for *Nod2* in the early development and composition of the intestinal microbiota^35^. Importantly, the authors showed that *NOD2* genotypes also influence the microbial composition in humans as the CD patients homozygous for the CD-associated *NOD2* allele exhibited differences in bacterial composition in non-inflamed ileal mucosa or in the feces compared to those homozygous for the wild type *NOD2* ^allele 35^. A more recent study also showed that CD patients homozygous for the *ATG16L1* risk allele showed impaired clearance of pathosymbionts with an altered bacterial composition in inflamed ileal tissue compared to those homozygous for the *ATG16L1* protective allele^49^. These studies highlight the preponderant role of autophagy in the control of gut microbiota homeostasis and to a larger extent in CD development.

The mechanism by which AIEC colonization modifies the gut microbiota composition in *Eif2ak4-*deficient mouse models remains uninvestigated. However, we can hypothesize that the colonization of AIEC might generate a specific “micro-environment” in the gut that allows the selection of a pro-inflammatory bacterial population. This action was indirect given by the fact that the gut microbiota modification in these genetically predisposed mouse models appeared numerous days after AIEC became undetectable in the feces. Our data are supported by the study by Chassaing and colleagues, which showed that a transient AIEC colonization induces an alteration in bacterial microbiota composition in germ-free *tlr5*^−/−^ mice^43^.

Although so far no *EIF2AK4* variant is associated with CD susceptibility^50^, studies have begun to show the importance of EIF2AK4 in CD. Indeed, an increase in the expression of EIF2AK4 in patients with CD compared to the controls has been reported^32–34^. Our group previously showed a defect in the activation of the EIF2AK4-EIF2A-ATF4 pathway under inflammatory condition in CD patients^30^. In mice, we showed that the mouse models with *Eif2ak4* deficiency exhibit increased susceptibility to AIEC infection with increased gut colonization by AIEC and increased AIEC-induced inflammation due to autophagy defect^30^ and gut dysbiosis upon a AIEC colonization, which might contribute to the development of chronic inflammation (data from the current study). These results together suggest that *Eif2ak4*-deficient mice could be considered as genetically predisposed mouse models to study the implication of AIEC in the etiopathogenesis of CD.

To summarize, this study showed that AIEC colonization leads to a modification of the gut microbiota composition in *Eif2ak4*-deficient mice. This may result in the selection of a pro-inflammatory bacterial population, thereby contributing to the development of chronic intestinal inflammation.

## Materials and Methods

### Mice

*eif2ak4*^−/−^ mice^51^ were kindly provided by Dr. D. Ron (Institute of Metabolic Science, Cambridge, UK). Heterozygote CEABAC10 transgenic (Tg) male mice^25^ were backcrossed for ten generations with 5-week-old female C57BL/6 mice (Charles River Laboratories). Tg mice at the tenth backcross were then crossed with *eif2ak4*^−/−^ mice to generate Tg/*eif2ak4*^+/+^ and Tg/*eif2ak4*^−/−^ mice. All mice were housed under specific pathogen-free conditions in the animal care facility at the University of Clermont Auvergne (Clermont-Ferrand, France).

### *In vivo* infection and bacterial colonization evaluation

At age of 12 weeks, *eif2ak4*^+/+^ and *eif2ak4*^−/−^ mice, or Tg/*eif2ak4*^+/+^ and Tg/*eif2ak4*^−/−^ mice were administered orally by gavage during three days (once per day) with either 10^9^ of ampicillin-erythromycin-resistant AIEC LF82 strain isolated from a CD patient^14^ or the rifampicin-resistant non-pathogenic *E*. *coli* K12 MG1655 strain. Bacterial persistence in the gut was evaluated every day post-infection. For this, fresh fecal samples (100–200 mg) were homogenized in PBS. After serial dilutions, fecal samples were plated on LB agar plates containing 50 μg/ml ampicillin and 20 μg/ml erythromycin (Euromedex, E002) to isolate LF82 bacteria, or containing 300 μg/ml rifampicin (Euromedex, 1059-B) to isolate *E*. *coli* K12 bacteria, and were incubated overnight at 37 °C. The number of bacteria was determined by counting the colony-forming units (CFU).

### Quantification of fecal lipocalin-2

The levels of the inflammatory marker lcn-2 were measured in feces by enzyme-linked immunosorbent assays (ELISA) as previously described^41^. For this, frozen fecal samples were reconstituted in PBS containing 0.1% Tween 20 (Euromedex, 2001-A) and were disrupted to obtain a homogenous fecal suspension. These samples were then centrifuged for 10 minutes at 12,000 rpm at 4 °C. Clear supernatants were collected and stored at −80 °C until analysis. Lcn-2 levels in the supernatants were measured using a mouse Duoset Lcn-2 ELISA kit (R&D Systems, USA) according to the manufacturer’s instructions.

### Microbiota composition analysis by Illumina Sequencing

DNA was extracted from frozen mouse fecal samples using NucleoSpin® Soil (Macherey-Nagel GmbH & Co, Germany) following manufacturer’s instructions. DNA samples were used for 16S rRNA gene sequencing using the Illumina technology as previously described^41^. Briefly, the 16S rRNA gene V4 variable region PCR primers F515/R806^52^ with barcode on the forward primer were used in a PCR using the HotStartTaq Plus Master Mix Kit (Qiagen, USA) under the following conditions: 94 °C for 3 minutes, followed by 28 cycles of 94 °C for 30 seconds, 53 °C for 40 seconds and 72 °C for 1 minute, after which a final elongation step at 72 °C for 5 minutes was performed. After amplification, PCR products were checked in 2% agarose gel to determine the success of amplification and the relative intensity of bands. All samples were pooled together in equal proportions based on their molecular weight and DNA concentrations. Pooled samples were purified using calibrated Ampure XP beads (Agencourt Bioscience Corporation, USA). Then the pooled and purified PCR product was used to prepare DNA library by following Illumina TruSeq DNA library preparation protocol. Sequencing was performed at MR DNA (www.mrdnalab.com, Shallowater, TX, USA) on a MiSeq following the manufacturer’s guidelines. Sequence data were processed using MR DNA analysis pipeline (MR DNA, Shallowater, TX, USA). In summary, sequences were joined, depleted of barcodes then sequences <150 bp were removed, sequences with ambiguous base calls were removed. The software package, Quantitative Insights Into Microbial Ecology (QIIME) was used for filtering and analysis of attained sequences. Chimeras were removed and Operational taxonomic units (OTUs) were generated by clustering at 3% divergence (97% similarity). Final OTUs were taxonomically classified using BLASTn against a curated database derived from GreenGenes^53^, RDPII and NCBI (www.ncbi.nlm.nih.gov, http://rdp.cme.msu.edu).

Singletons and any OTUs present in all samples <0.1% were removed before further analyses. Subsequent analyses of diversity were performed at a depth of 20,000 sequences per sample. Beta diversity was performed with QIIME using a Principal Coordinate Analysis (PCoA), measuring dissimilarities at phylogenetic distances based on weighted UniFrac analysis. QIIME was also used to provide the relative abundance of bacterial groups at different taxonomic levels between each group of mice.

### Quantification of target bacterial group by qPCR

DNA extracted from frozen mouse fecal samples as described above was used for qPCR to quantify target bacterial group. Each qPCR reaction mixture consisted of iQ SYBR Green Supermix (Biorad), 0.25 µM of each primer and 10 ng of extracted DNA. To detect AIEC LF82, primers binding to the LF82-specific *pMT* gene (forward 5′-CCATTCATGCAGCAGCTCTTT-3′ and reverse 5′-ATCGGACAACATTAGCGGTGT-3′) were used^37^. A region of the 16S rRNA gene of all bacteria was amplified using the universal primers (Forward: 5′-ACTCCTACGGGAGGCAG-3′ and reverse 5′-GACTACCAGGGTATCTAATCC-3′)^54^. Amplification programs included an initial denaturation at 95 °C for 10 min followed by 40 cycles consisting of denaturation at 95 °C for 30 sec, annealing at 55 °C (all bacteria) and 60 °C (LF82-specific gene *pMT*) for 30 sec and extension at 72 °C for 5 min. Data were normalized to total bacterial. Fold-induction was calculated using the ΔΔCt method as follows: ΔΔCt = (Ct_*pMT*_ − Ct_total bacteria_)_group 2_ − (Ct_*pMT*_ − Ct_total bacteria_)_group 1_, and the final data were derived from 2^−ΔΔCt^_._

### Ethical statement

Animal protocols were carried out in strict accordance with the recommendations of the Guide for the Care and Use of Laboratory Animals of the Université Clermont-Auvergne, Clermont-Ferrand, France and were approved by the Ethical Committee for Animal Experimentation of the Department of Auvergne (Comité d’éthique en matière d’expérimentation animale CEMEA Auvergne, registered under the number C2EA - 02) and the Ministère de l’Education Nationale, de l’Enseignement Supérieur et de la Recherche (APAFIS#2500-20 15070710163594).

### Statistical analysis

For bacterial CFUs, lcn-2 levels and relative abundance of bacterial genus data, values were expressed as means ± SEM. Statistical analysis was performed using one-way ANOVA test followed by Bonferroni’s post-hoc comparisons with GraphPad Prism version 5.01 software (GraphPad Software, San Diego, CA). PCoA data of mouse groups were compared, and statistical significance of clustering was determined using Permanova method using QIIME software package. A *P* value less than 0.05 was considered statistically significant. *P < 0.05; **P ≤ 0.01; ***P ≤ 0.001; ****P ≤ 0.0001.

## Electronic supplementary material

Supplementary figures
