# Supplementary material for: AIEC infection triggers modification of gut microbiota composition in genetically predisposed mice, contributing to intestinal inflammation
Source: Sci Rep. 2018 Aug 17;8:12301. doi: 10.1038/s41598-018-30055-y (PMC6098085; doi:10.1038/s41598-018-30055-y)
Supplement: Supplementary file 1 — Supplementary figures [file 41598_2018_30055_MOESM1_ESM.pdf]

AIEC infection triggers modification of gut microbiota composition in genetically predisposed mice, contributing to intestinal inflammation

Alexis Bretin[1], Cécily Lucas[1], Anaïs Larabi[1], Guillaume Dalmasso[1], Nicolas Barnich[1], Richard Bonnet[1,2], Hang Thi Thu Nguyen[1,*]

[1]M2iSH, UMR 1071 Inserm, University of Clermont Auvergne, INRA USC 2018, Clermont-Ferrand 63001, France.

[2]Centre Hospitalier Universitaire, Clermont-Ferrand 63001, France

[*]**Address correspondence to**: Hang Nguyen, PhD. M2iSH, UMR 1071 Inserm, University of Clermont Auvergne, INRA USC 2018, Clermont-Ferrand 63001, France;

Phone: 33 4 73 17 83 72. Fax: 33 4 73 17 83 71. E-mail: hang.nguyen@uca.fr or hang.nguyen@udamail.fr

Running head: AIEC induce gut dysbiosis, contributing to inflammation

A

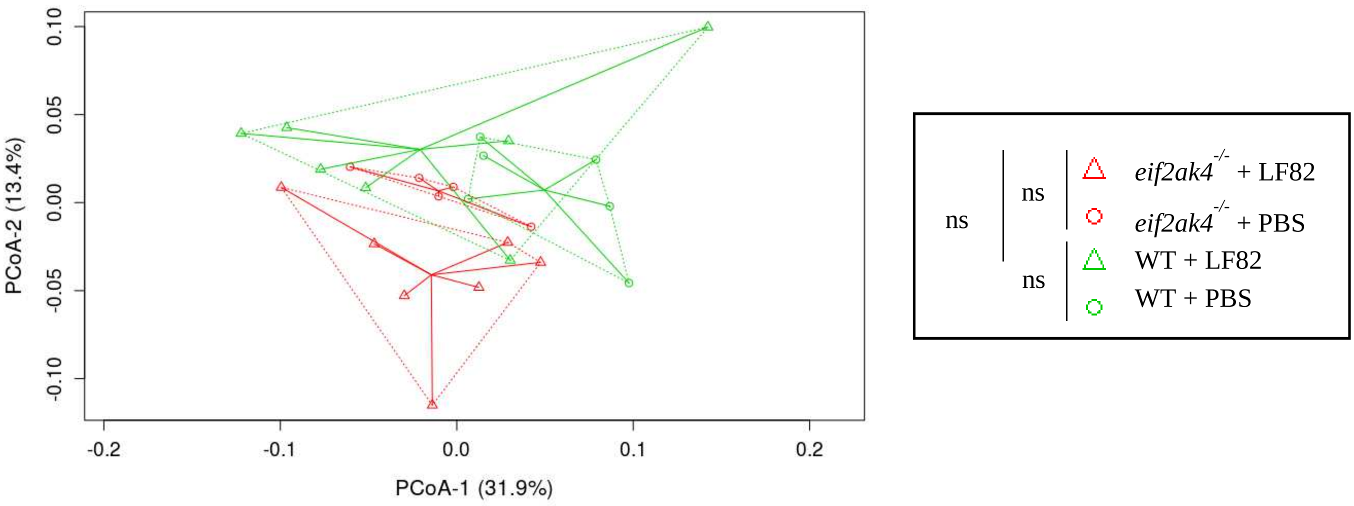

B

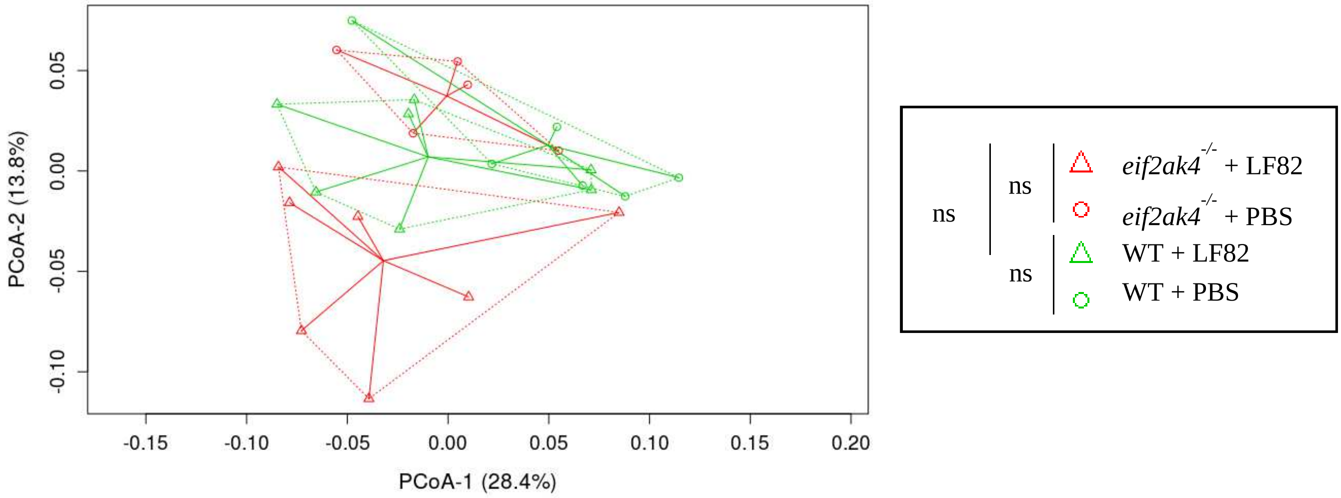

Figure S1. Transient colonization of *eif2ak4*$^{+/+}$ and *eif2ak4*$^{-/-}$ mice with AIEC does not result in modification of the gut microbiota composition at day 1 and 4 post-infection. *eif2ak4*$^{+/+}$ and *eif2ak4*$^{-/-}$ mice were challenged by oral gavage for 3 days (once per day) with PBS (N=6 mice per group) or with 10^9 CFU of the AIEC LF82 strain (N=7 mice per group). Feces were collected at day 1 (**A**) and 4 (**B**) post-infection for the analysis of the bacterial microbiota composition based on Illumina pyrosequencing of the 16S rRNA gene. Mouse fecal bacterial communities were clustered using PCoA of the weighted UniFrac distance matrix. PCoA-1 and PCoA-2 were plotted, and the percentage of the variation explained by the plotted principal coordinates was indicated in the Y-X-axis labels. Comparison between *eif2ak4*$^{+/+}$ mice + PBS, *eif2ak4*$^{+/+}$ mice + LF82, *eif2ak4*$^{-/-}$ mice + PBS and *eif2ak4*$^{-/-}$ mice + LF82 groups at day 1 (**A**) and 4 (**B**) post-infection. Groups were compared using permanova method. Ns, not significant.

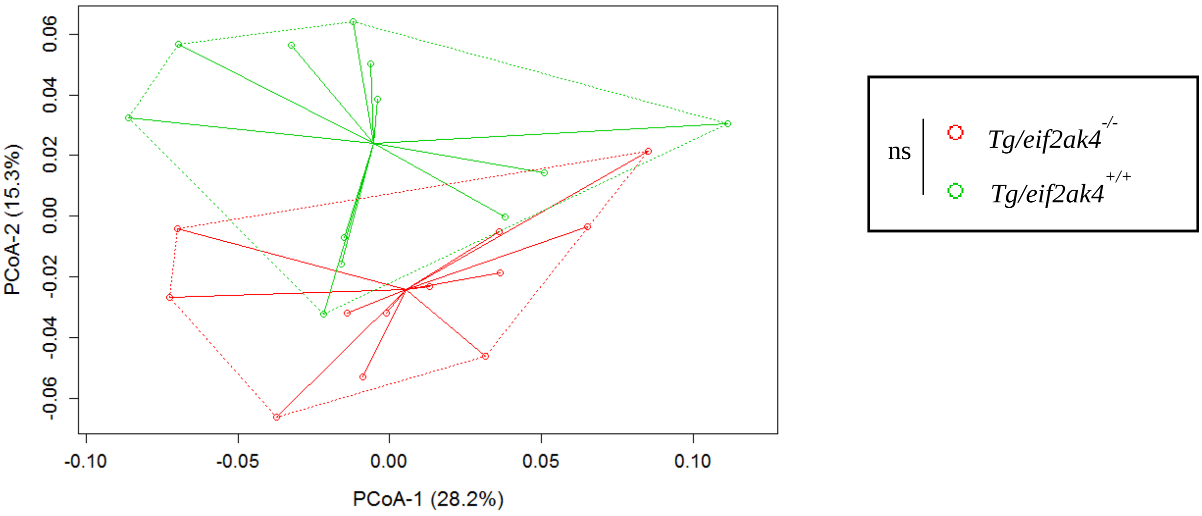

Figure S2: *Eif2ak4* gene deficiency has no impact on the gut microbiota composition of CEABAC10 transgenic (Tg) mice.

Tg/eif2ak4$^{+/+}$ (N=13) and *Tg/eif2ak4*$^{-/-}$ (N=13) mice were placed in specific-pathogen free housing conditions. Feces were collected from 12-week old mice for the analysis of the bacterial microbiota composition based on Illumina pyrosequencing of the 16S rRNA gene. Mouse fecal bacterial communities were clustered using PCoA of the weighted UniFrac distance matrix. PCoA-1 and PCoA-2 were plotted, and the percentage of the variation explained by the plotted principal coordinates was indicated in the Y-X-axis labels. Groups were compared using permanova method. Ns, not significant.

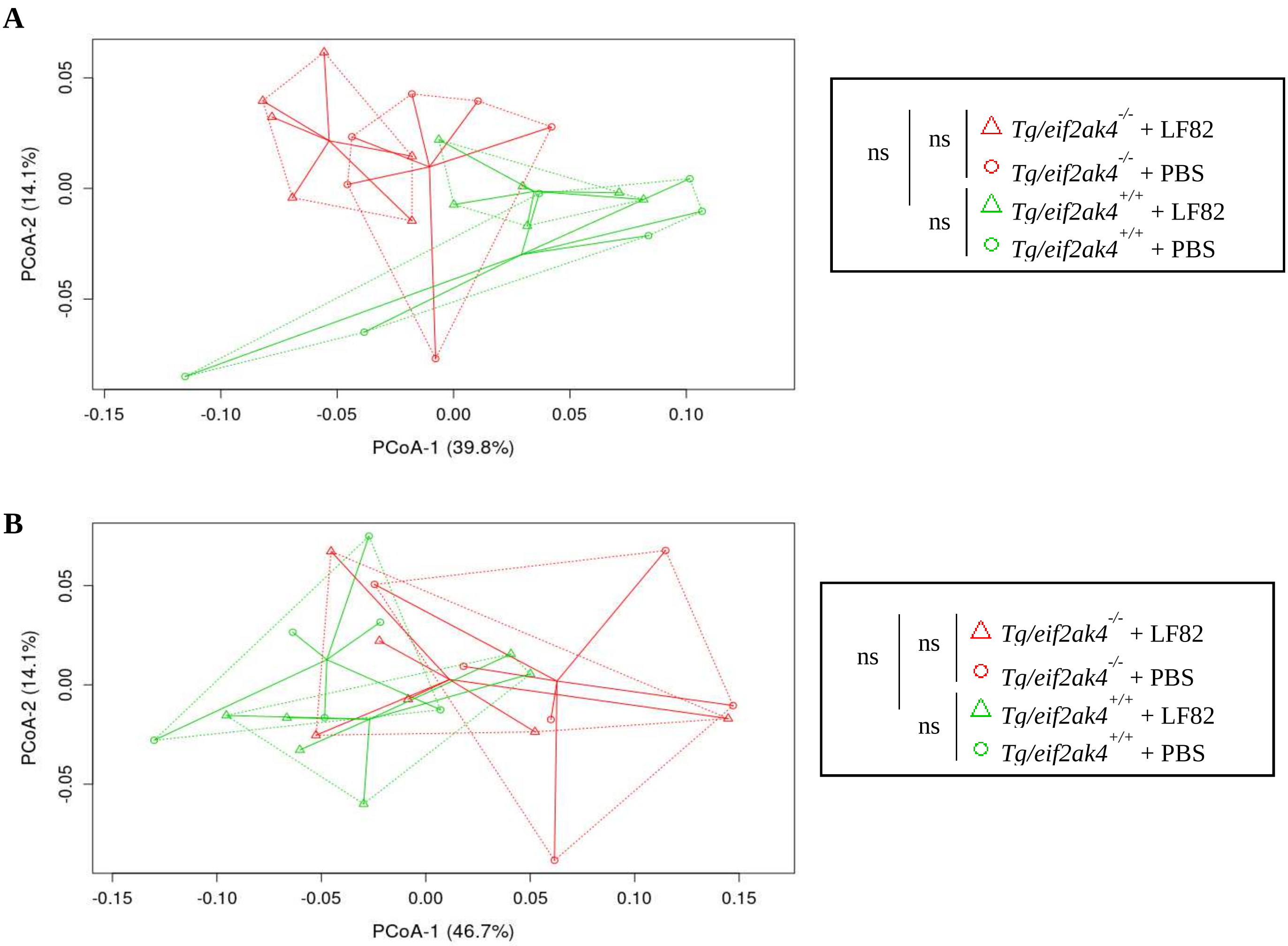

Figure S3: Transient colonization of CEABAC10 transgenic (Tg) mice with *Eif2ak4* gene deficiency or not with AIEC does not result in modification of the gut microbiota composition at day 1 and 4 post-infection.

Tg/*eif2ak4*$^{+/+}$ and Tg/*eif2ak4*$^{-/-}$ mice were challenged by oral gavage for 3 days (once per day) with PBS (N=6 mice per group) or with 10^9 CFU of the AIEC LF82 strain (N=6 mice per group). Feces were collected at day 1 (**A**) and 4 (**B**) post-infection to analyze the bacterial microbiota composition based on Illumina pyrosequencing of the 16S rRNA gene. Mouse fecal bacterial communities were clustered using PCoA of the weighted UniFrac distance matrix. PCoA-1 and PCoA-2 were plotted, and the percentage of the variation explained by the plotted principal coordinates was indicated in the Y-X-axis labels. Comparison between Tg/*eif2ak4*$^{+/+}$ mice + PBS, Tg/*eif2ak4*$^{+/+}$ mice + LF82, Tg/*eif2ak4*$^{-/-}$ mice + PBS and Tg/*eif2ak4*$^{-/-}$ mice + LF82 groups at day 1 (**A**) and 4 (**B**) post-infection. Groups were compared using permanova method. Ns, not significant.
